# Analysis of neonatal clinical trials with twin births

**DOI:** 10.1186/1471-2288-9-12

**Published:** 2009-02-26

**Authors:** Michele L Shaffer, Allen R Kunselman, Kristi L Watterberg

**Affiliations:** 1 Department of Public Health Sciences, Penn State College of Medicine, Hershey, PA, USA; 2 Department of Pediatrics, Penn State College of Medicine, Hershey, PA, USA; 3 Department of Pediatrics/Neonatology, University of New Mexico School of Medicine, Albuquerque, NM, USA

## Abstract

**Background:**

In neonatal trials of pre-term or low-birth-weight infants, twins may represent 10–20% of the study sample. Mixed-effects models and generalized estimating equations are common approaches for handling correlated continuous or binary data. However, the operating characteristics of these methods for mixes of correlated and independent data are not well established.

**Methods:**

Simulation studies were conducted to compare mixed-effects models and generalized estimating equations to linear regression for continuous outcomes. Similarly, mixed-effects models and generalized estimating equations were compared to ordinary logistic regression for binary outcomes. The parameter of interest is the treatment effect in two-armed clinical trials. Data from the National Institute of Child Health & Human Development Neonatal Research Network are used for illustration.

**Results:**

For continuous outcomes, while the coverage never fell below 0.93, and the type I error rate never exceeded 0.07 for any method, overall linear mixed-effects models performed well with respect to median bias, mean squared error, coverage, and median width. For binary outcomes, the coverage never fell below 0.90, and the type I error rate never exceeded 0.07 for any method. In these analyses, when randomization of twins was to the same treatment group or done independently, ordinary logistic regression performed best. When randomization of twins was to opposite treatment arms, a rare method of randomization in this setting, ordinary logistic regression still performed adequately. Overall, generalized linear mixed models showed the poorest coverage values.

**Conclusion:**

For continuous outcomes, using linear mixed-effects models for analysis is preferred. For binary outcomes, in this setting where the amount of related data is small, but non-negligible, ordinary logistic regression is recommended.

## Background

### Introduction

Neonatal studies involving singletons and twin births pose a unique correlated data problem. Data from singletons and twins whose siblings are not included in the study (unmatched twins) meet the basic assumption of independence, while the remaining complete twin births have a hierarchical structure. In the absence of twin births, classical statistical techniques are valid and appropriate. In the absence of singletons, hierarchical methods that account or adjust for the nested structure can be applied. Failing to account for the hierarchical structure within complete twin births may impact the estimate of target sample size and the precision of treatment effect estimates and/or decisions regarding treatment efficacy. Therefore, these effects may need to be quantified and accounted for in studies that involve both singletons and twin births. If the proportion of infants from complete twin pairs is small, e.g., less than 5%, there may be minimal impact. Additionally, methods that account for correlation are computationally more difficult and may fail if too little data are available to adequately model or appropriately adjust for correlation. Thus, in some circumstances, it is possible classical statistical techniques may be sufficient or preferred.

In the very low birth weight group (501–1500 g), 20% or more of infants are products of multiple gestations, primarily twins, due in part to the increasing number of pregnancies resulting from assisted reproductive technology [1,2]. It is unknown whether twin outcomes are more similar than unrelated individuals due to genetic similarity, or more different than unrelated individuals because of increased illness severity of one twin, usually the "B", or second twin.

While ophthalmic studies also can produce data where a small proportion of observations are correlated, there are important distinctions between within-birth correlation and between-eye correlation. All sets of eyes share identical genetic information, whereas only identical twins have identical genetic makeup. Before the introduction of assisted reproductive technology, 25–30% of twin births were monozygotic [3]. Taking into consideration reproductive technology, less than 10% of twin births in the very low birth weight population are expected to be monozygotic. Eyes from the same subject are commonly expected to show greater similarity than eyes from unrelated subjects in reaction to a particular stimulus or disease [4-7]. However, while monozygotic twins may be expected to show greater similarity, for dyzygotic twins other factors such as sex, birth weight discordance, and like-sex/unlike-sex pairing may be more important [8-10]. Thus, monozygotic twins represent one end of the spectrum of genetic similarity, rather than a representation of the entire population of twins. It also should be noted that zygosity is not always known at birth and is not routinely collected for neonatal studies; however, classical twin studies do assume different within-birth correlations for monozygotic and dyzygotic twins [11].

### Motivating example: The IVIG trial

The Randomized Clinical Trial of Intravenous Immune Globulin to Prevent Neonatal Infection in Very-Low-Birth-Weight (501–1500 g) Infants (IVIG) trial was a multi-center, two-phase controlled trial of 2416 infants conducted by the NICHD Neonatal Research Network [12]. Infants were randomized within 72 hours of birth to an intravenous immune globulin (IVIG) group (1204) or a control group (1212), stratified by birth weight (501–1000, 1001–1550 g) and clinical center. Control infants were given placebo infusions during phase 1 of the study (n = 623) but were given no infusions during phase 2 (n = 589). Infants weighing 501 to 1000 g at birth were given 900 mg of immune globulin per kg of body weight, and infants weighing 1001 to 1500 g at birth were given 700 mg per kg within 24 hours of randomization. The infusions were repeated every 14 days until the infants weighed 1800 g, were transferred to another center, died, or were sent home from the hospital. Serum IgG levels (in mg/dL) were measured at enrollment and before each infusion. The trial reported a multiple gestation rate of 16% with twins randomized independently as individuals; pregnancies of three or more fetuses were excluded from the study. Thirteen percent of the infants enrolled in the study were twins whose siblings also were enrolled. The primary outcome was the development of confirmed nosocomial infection, including septicemia, meningitis, or urinary tract infections, during the first 120 days of life. Secondary outcomes included mortality and neonatal morbidity, assessed in terms of duration of ventilator support, frequency of bronchopulmonary dysplasia, and duration of hospitalization.

### Analytic approaches

Gates and Brocklehurst compared methods of analysis for binary outcomes in randomized controlled trials where the unit of randomization was the mother (or equivalently, birth) [13]. The methods compared were 1) assuming independence among infants, 2) analyzing outcomes per mother, 3) randomly selecting one infant from each multiple birth for inclusion in the analysis, and 4) cluster trial methods. Cluster trial methods were found to be advantageous for outcomes that are most appropriately analyzed by infant rather than mother. However, these methods cannot be applied in clinical trials where infants from multiple pregnancies are randomized independently or to opposite treatments and do not allow adjustment for covariates.

Gates and Brocklehurst suggested that more complicated statistical techniques such as mixed-effects models and generalized estimating equations (GEE) are more difficult to apply and understand [13]; however, both methods are readily available in several comprehensive statistical packages, including SAS (SAS Institute Inc., Cary, North Carolina, USA), R (R Foundation for Statistical Computing, Vienna, Austria), Stata (StataCorp LP, College Station, Texas, USA), and SPSS (SPSS Inc., Chicago, Illinois, USA). Both methods allow adjustment for covariates and permit consideration of additional randomization schemes.

Linear mixed-effects models (LMEM) introduced by Laird and Ware allow for both correlation of outcomes and heterogeneity of variance for continuous outcomes [14]. The structure of the correlation between observations is modeled through the specification of a "within-subject," or here within-birth correlation matrix. For binary outcomes, a generalized linear mixed model (GLMM) can be used by adding a random term to an ordinary logistic regression model which induces a within-birth correlation [15,16]. GLMM have been reviewed more recently by Molenberghs and Verbeke and Hedeker and Gibbons [17,18].

Rather than explicitly modeling the within-birth correlation, GEE can be used to obtain a robust sandwich estimator of the standard error for the treatment effect, which can be used for hypothesis testing or confidence interval construction [19,20]. GEE are applied using a working correlation matrix to account for correlated outcomes and yield consistent, but not necessarily efficient, estimators of the model parameters. The within-birth correlation is treated as a nuisance, or something to be adjusted for, but is not of primary interest.

One difference between the mixed-effects models and marginal models, including GEE, is that the treatment effects estimated from the marginal models show the effect of treatment averaged over the population of subjects, while mixed-effects models provide conditional estimates obtained by conditioning on the random effect. There is no difference in scale for LMEM and the corresponding marginal models, so this distinction is often ignored; however, there is a difference in scale for nonlinear models, such as the logistic regression model [18]. The estimates obtained from the GLMM formed by adding a random effect to the ordinary logistic regression model can be marginalized using the formula

(1) $${\hat{\beta }}_{M}\approx {\hat{\beta }}_{RE}/{\left(c^{2}{\hat{\tau }}^{2}+1\right)}^{1/2}$$

where ${\hat{\beta }}_{M}$ is the marginalized parameter estimate, ${\hat{\beta }}_{RE}$ is the parameter estimate from the GLMM which includes a random effect, ${\hat{\tau }}^{2}$ is the estimated variance of the random effect, and $c=16\sqrt{3}/(15\pi )$[21].

While these established methods exist for handling correlated data, it is unknown which methods are optimal for studies that may have as little as 10% of the individuals related.

For ophthalmic data, Glynn and Rosner compared ordinary analysis of variance (ANOVA) and a special case of the linear mixed-effects model for continuous outcomes and logistic regression and GEE for binary outcomes using two observational studies where all patients contributed outcomes from both eyes [22]. Additionally, 50% of subjects had one eye randomly deleted from each of the data sets to examine the effect of having a smaller proportion of related data. Confidence intervals were appropriately wider and standard errors larger when the correlation was taken into account. Glynn and Rosner presented further comparisons of logistic regression and GEE for binary outcomes under six conditions based on one of the previously considered observational studies [23]. One thousand simulated data sets were used for each condition. Type I error rates and power for logistic regression were found to be inferior to GEE. While eyes from the same subject were assumed to be more similar than unrelated eyes, the range of correlation covered by the six simulated data sets was not clear.

Leite and Nicolosi proposed a weighted logistic regression method to account for the correlation between eyes for binary outcomes [24]. The weighted approach, which is similar to GEE in that variance is inflated by a factor that is a function of the between-eye correlation, was compared to ordinary logistic regression. Since eyes from the same subject were assumed to be more similar than eyes from unrelated subjects, simulations studies were conducted across a range of positive correlations (0.1–0.9), and the correlation was allowed to differ by an exposure (or treatment) that had only two levels. Two sample sizes were considered, 100 per group and 40 per group. The unit of randomization was the subject so that both eyes received the same treatment. One thousand simulated data sets were used for each case. The simulations showed inflated type I error rates for ordinary logistic regression, while the weighted approach performed well over the range or correlations considered.

The purpose of this paper was to determine if and when mixed-effects models or GEE were operationally superior to classical statistical techniques that assume independence for two-armed, neonatal clinical trials with continuous or binary outcomes, while taking into account the randomization scheme.

## Methods

Simulation studies were conducted using SAS Version 9 to operationally compare linear regression (one-way ANOVA when no covariates are present), LMEM, and GEE for continuous outcomes and ordinary logistic regression, GLMM, and GEE for binary outcomes in two-armed clinical trials.

With *i *= 1, ..., *n *total subjects, the TTEST procedure was used to fit regression models of the form

(2) *y*_*i *_= *α *+ *X_i_β *+ *ε*_*i*_

where *y*_*i *_is the response for subject *i*, *α *is the intercept parameter, *X*_*i *_is the treatment group indicator variable for subject *i*, *β *is the treatment effect parameter, and *ε*_*i *_is the error for subject *i*. Using restricted maximum likelihood estimation [25], the MIXED procedure was used to fit LMEM of the form

(3) **y**_*i *_= **X**_*i*_***β ***+ **ε**_*i*_

where **y**_*i *_is the *n*_*i *_× 1 response variable for birth *i*, **X**_*i *_is the *n*_*i *_× 2 design matrix for birth *i*, **β **is the 2 × 1 parameter vector which includes the intercept and one treatment effect parameter, and **ε**_*i *_is the *n*_*i *_× 1 error vector. **ε**_*i *_is assumed to be normally distributed with zero mean and a compound symmetric variance-covariance matrix. The procedure LOGISTIC, specifying the maximum likelihood method based on iteratively reweighted least squares [15], was used to fit ordinary logistic regression models of the form

(4) $$\log\left[\frac{P(y_{i}=1)}{1-P(y_{i}=1)}\right]=\alpha +X_{i}\beta +\varepsilon_{i}$$

where *y*_*i *_is the binary response for subject *i *that can take the value 0 or 1, *α *is the intercept parameter, *X*_*i *_is the treatment group indicator variable for subject *i*, *β *is the treatment effect parameter, and *ε*_*i *_is the error for subject *i*. Using marginal maximum likelihood [26], the NLMIXED procedure was used to fit GLMM of the form

(5) $$\log\left[\frac{P(y_{ij}=1)}{1-P(y_{ij}=1)}\right]=\alpha +X_{ij}\beta +\nu_{i}+\varepsilon_{ij}$$

where *y*_*ij *_is the binary response for subject *j *within birth *i *that can take the value 0 or 1, *α *is the intercept parameter, *X*_*ij *_is the treatment group indicator variable for subject *j *within birth *i*, *β *is the treatment effect parameter, *ε*_*ij *_is the error for subject *j *within birth *i*, and *ν*_*i *_is the random birth effect, assumed to be normally distributed with mean 0 and variance *τ*^2^. The GENMOD procedure was used for the GEE approach, assuming an exchangeable structure for the working correlation [21].

A variety of scenarios were considered, varying the randomization scheme for complete twin pairs, sample size, proportion of infants from complete twin pairs, correlation within twin births, and effect size. Values of parameters used in the simulation studies are shown in Table 1, and 10,000 data sets were generated for each simulation case. Operating characteristics collected included type I error rate, coverage, median bias, mean squared error, and median width. All tests were conducted at the 0.05 level of significance and 95% confidence intervals were computed.

**Table 1:** Parameter values for simulation studies

Simulation parameter	Values
Randomization of complete twin pairs	Same arm, independently, opposite arms
Total sample size	250, 500
Proportion of infants from complete twin pairs	0.1, 0.2
Continuous outcomes	
Standardized effect size	0, 0.5, 1, 1.5
Within-birth correlation	0, 0.25, 0.5
Binary outcomes	
Treatment effect, measured on log scale	-1, -0.5, 0
Log odds ratio measuring within-birth correlation	-2, -1.5, -1, -0.5

For continuous outcomes singleton and unmatched twin data were generated using a normal distribution. The mean and variance of the normal distribution were determined by the standardized effect sizes shown in Table 1. Complete twin pair data were generated using a bivariate normal distribution for which the correlation is set by the correlation assumed within twin pairs.

For binary data singleton and unmatched twin data were generated using a binomial distribution. Complete twin pair data were generated using Rosner's extension of the beta-binomial model [27]. Conditional on the treatment assignment, the marginal probability of an infant from a complete twin pair having an event was the same as the probability of a singleton or unmatched twin having an event.

A permuted blocks approach was used for randomization. Treatment assignments for complete twin pairs depended upon the randomization scheme. When twins were randomized as individuals, the process was the same as for singletons and unmatched twins. When complete twin pairs were always assigned to the same treatment or opposite treatments, a treatment assignment was generated for a single twin in the pair, automatically determining the second twin's assignment.

If convergence problems were encountered for the GLMM or GEE approaches with binary data, an ordinary logistic regression analysis was performed. In order to make the results comparable for the GLMM approach, the parameter estimates were marginalized using equation (1).

## Results and discussion

### Simulation studies

Selected simulation results for treatment effect hypothesis testing for continuous outcomes are shown in Additional file 1. Generally, the median bias was small, regardless of the values of simulation parameters considered. Mean squared error decreased as the sample size increased; otherwise, there was little change in mean squared error with regard to the other simulation parameters. When there was no within-birth correlation, ANOVA and LMEM performed similarly with regard to coverage and median width, regardless of the randomization method, sample size, proportion of twins, and effect size. GEE showed poorer coverage than either ANOVA or LMEM when there was no within-birth correlation, producing confidence intervals that had smaller median width. When within-birth correlation was present and randomization of twins was to the same arm, GEE tended to be a compromise between ANOVA and LMEM with regard to coverage and median width. LMEM showed the best performance with regard to coverage and median width. When within-birth correlation was present and randomization of twins was done independently or to opposite arms, GEE showed the poorest performance with respect to coverage. When randomization of twins was done independently, LMEM showed the best performance for all but the largest sample size and within-birth correlation. However, the differences between ANOVA and LMEM in these cases were modest. When randomization of twins was to opposite treatment arms, LMEM provided coverage values closer to the nominal value, while producing confidence intervals with smaller median width. ANOVA produced confidence intervals that were too wide with coverage beyond the nominal level. Generally, no method performed very poorly; the coverage never fell below 0.93, and the type I error rate never exceeded 0.07. The worst performance values for estimation of within-birth correlation for continuous outcomes are shown in Table 2. Overall, LMEM provided a less biased estimate of within-birth correlation than GEE.

**Table 2:** Worst performance values for estimation of within-birth correlation for continuous outcomes

		Operating characteristic	
		
Randomization	Method	Mean bias	Median bias
Same	LMEM	-0.014	0.019
	GEE	0.053	0.050
Independent	LMEM	-0.020	0.014
	GEE	0.047	0.042
Opposite	LMEM	-0.015	0.020
	GEE	0.060	0.059

Mathematically, one can show under simplified conditions for continuous data that the mean and variance of the estimated treatment effect, ${\bar{Y}}_{T}-{\bar{Y}}_{C}$, for a two-armed clinical trial are given by

$$\begin{array}{c} E({\bar{Y}}_{T}-{\bar{Y}}_{C})=\mu_{T}-\mu_{C},\\ V({\bar{Y}}_{T}-{\bar{Y}}_{C})=V({\bar{Y}}_{T})+V({\bar{Y}}_{C})-2Cov({\bar{Y}}_{T},\bar{Y}c)\\ =\{\begin{array}{l} \sigma^{2}/n+\sigma^{2}/n-0=2\sigma^{2}/n,\text{ when }\rho =\text{0 regardless of randomization,}\\ \sigma^{2}/n+\sigma^{2}/n-2p_{Twin}\rho /n=2\sigma^{2}/n-2p_{Twin}\rho /n,\;\\ \text{when randomized to opposite arms,}\\ (\sigma^{2}/n+p_{Twin}\rho /n)+(\sigma^{2}/n+p_{Twin}\rho /n)-0=2\sigma^{2}/n+2p_{Twin}\rho /n,\;\\ \text{when randomized to the same arm,}\\ (\sigma^{2}/n+p_{Twin}\rho /2n)+(\sigma^{2}/n+p_{Twin}\rho /2n)-p_{Twin}\rho /n=2\sigma^{2}/n,\;\\ \text{when randomized independently,} \end{array} \end{array}$$

where *σ*^2 ^is the variability of the outcome variable, *n *is the sample size per group, *p*_*Twin *_is the proportion of infants from complete twin pairs, and *ρ *is the correlation within twin births. Simplifying assumptions include treatment allocation that is perfectly balanced, equal numbers of complete twin pairs within treatment arms, and treatment allocation in the proportions 0.25, 0.25, and 0.5 for control-control, treatment-treatment, and control-treatment, respectively, when twins are randomized independently. Thus, the over- or under- estimation of the variability is at most 2/*n*, which is small when *n *is large.

Selected simulation results for treatment effect hypothesis testing for binary outcomes are shown in Additional file 2. Regardless of the simulation parameters, there was little difference between ordinary logistic regression and GLMM with respect to median bias and mean squared error. When randomization of twins was to the same treatment arm, GEE tended to produce estimates with greater median bias (in absolute terms) and mean squared error; however, this pattern did not persist when randomization was done independently or to opposite treatment arms. Ordinary logistic regression showed the best performance with respect to coverage when randomization of twins was to the same treatment arm, and the sample size was 250. When the sample size was 500, GLMM showed better performance, but ordinary logistic regression still performed well. There were cases when randomization of twins was to the same treatment arm where GEE produced wider intervals with lower coverage, which is consistent with the bias finding. GLMM showed the poorest performance with respect to coverage, producing confidence that had smaller median width, when randomization of twins was done independently or to opposite treatment arms, regardless of the other simulation parameters. Ordinary logistic regression showed the best performance with respect to coverage when twins were randomized independently, while the median width was not markedly different from the intervals produced by GEE. When randomization of twins was to opposite treatment arms, ordinary logistic regression and GEE performed similarly with respect to coverage and median width. No method performed very poorly; the coverage never fell below 0.90, and the type I error rate never exceeded 0.07.

When using GLMM, the intraclass correlation coefficients (ICCs) were estimated using

(6) $${\hat{\tau }}^{2}/({\hat{\tau }}^{2}+\pi^{2}/3)$$

which is applicable for a logistic model with normally distributed random effects [18].

The median ICCs were 0, and the mean ICCs were very small. When using GEE, the mean and median correlations estimated using Pearson residuals were negative. Thus, neither approach provided a useful measure of within-birth correlation for twin pairs.

### The IVIG trial revisited

Analyses of day 14 IgG levels using ANOVA, LMEM, and GEE and separated by birth weight group are shown in Table 3. Overall, within birth weight groups the estimated treatment differences (IVIG group – control group) were similar. Given the relatively large treatment differences in both birth weight groups, the small changes in standard error did not affect the inference. In all cases, infants treated with IVIG showed significantly higher levels of IgG at day 14.

**Table 3:** Analysis of day 14 IgG levels (mg/dl) for the IVIG trial

Birth weight stratum	Analysis	Estimated correlation	Estimated treatment difference	Standard error	95% Confidence interval
501 to 1000 g	ANOVA	N/A	194.05	10.47	(173.50,214.61)
	LMEM	0.41	194.24	10.42	(173.78,214.71)
(n = 743 babies)	GEE	0.44	194.34	10.37	(174.02,214.65)
1001 to 1500 g	ANOVA	N/A	177.23	10.21	(157.20,197.26)
	LMEM	0.54	179.79	10.03	(160.11,199.47)
(n = 1267 babies)	GEE	0.36	178.74	10.06	(159.03,198.46)

Randomization was done independently for twins. The multiple gestation rates were 15% and 16% for the 501 to 1000 g babies and 1001 to 1500 g babies, respectively.

Analyses of bronchopulmonary dysplasia using ordinary logistic regression, GLMM, and GEE and separated by birth weight group are shown in Table 4. The estimates from GLMM have been marginalized using (1) to allow direct comparisons with the other results. Overall, within birth weight groups the estimated odds ratios were similar, and the small changes in the standard error did not affect the inference. It is interesting to note that the standard error did not change within birth weight group when comparing the ordinary logistic regression and GEE analyses; however, the odds ratio did change. In all cases, infants treated with IVIG had lower odds of bronchopulmonary dysplasia, although these results were not statistically significant.

**Table 4:** Analysis of bronchopulmonary dysplasia for the IVIG trial

Birth weight stratum	Analysis	Estimated correlation	Odds ratio	Standard error	95% Confidence interval
501 to 1000 g	Logistic	N/A	0.912	0.122	(0.702,1.185)
	GLMM	0.438	0.898	0.127	(0.649,1.146)
(n = 903 babies)	GEE	0.361	0.901	0.122	(0.691,1.175)
1001 to 1500 g	Logistic	N/A	0.944	0.155	(0.683,1.303)
	GLMM	0.537	0.923	0.138	(0.651,1.194)
(n = 1509 babies)	GEE	0.557	0.882	0.155	(0.626,1.244)

Randomization was done independently for twins. The multiple gestation rates were 15% and 16% for the 501 to 1000 g babies and 1001 to 1500 g babies, respectively.

## Conclusion

For continuous outcomes, using LMEM is recommended in comparison to either ANOVA or GEE, taking into consideration the following operating characteristics: median bias, mean squared error, coverage, and median width. Additionally, LMEM provide a measure of the within-birth correlation which may be of clinical interest. These results show there is little penalty to estimating a correlation within twin births, even when none is present. Given that we only need to estimate one additional parameter for the within-birth variance-covariance matrix, this result is not unexpected. Also, the mechanism of randomization does not markedly change the operating characteristics of LMEM. As the differences in variability of the estimated treatment effect for the three randomization schemes considered is small when the sample size is large, this result is logical given the sample sizes considered.

For binary outcomes, overall in the setting of these analyses were the amount of related data is small, but non-negligible, ordinary logistic regression is recommended in comparison to either GLMM or GEE. While these results may appear inconsistent with the results of the simulations for continuous outcomes, the relative amount of information available to estimate the correlation within twin births is less with binary outcomes than with continuous outcomes. Thus, one explanation is that the estimates of treatment effects obtained by ignoring the correlation within twin births and using ordinary logistic regression are better than estimating treatment effects in the presence of a poor estimate of correlation. Additionally, as estimates from GLMM require marginalization in order to make them comparable to the estimates derived from ordinary logistic regression and GEE, the confidence intervals considered were estimated using delta method. There may be operationally superior methods of deriving confidence intervals that would improve the performance of GLMM.

In the absence of covariates, LMEM are recommended for the analysis of continuous outcomes and in this limited setting ordinary logistic regression for binary outcomes. Future work includes sensitivity analyses to investigate the impact of adding covariates such as sex and birth weight.

## Competing interests

The authors declare that they have no competing interests.

## Authors' contributions

MS developed and participated in the conduct of the simulation studies and drafted the manuscript. AK participated in the conduct of the simulation studies. KW provided clinical expertise in selecting outcomes from the IVIG trial database to help design the simulations studies. All authors read and approved the final manuscript.

## Pre-publication history

The pre-publication history for this paper can be accessed here:

http://www.biomedcentral.com/1471-2288/9/12/prepub

## Supplementary Material

Additional file 1 **Supplementary Table**1. Selected simulation results for treatment effect hypothesis testing for continuous outcomes. Click here for file

Additional file 2 **Supplementary Table**2. Selected simulation results for treatment effect hypothesis testing for binary outcomes. Click here for file

## References

[B1] Schieve LA Meikle SF Ferre C Peterson HB Jeng G Wilcox LS Low and very low birth weight in infants conceived with use of assisted reproductive technology New England Journal of Medicine 2002 346 10 731 737 10.1056/NEJMoa010806 11882728

[B2] Martin JA Hamilton BE Sutton PD Ventura SJ Menacker F Munson ML Births: final data for 2002 National Vital Statistics Reports 2003 52 10 1 113 14717305

[B3] Luke B What is the influence of maternal weight gain on the fetal growth of twins? Clinical Obstetrics & Gynecology 1998 41 1 56 64 10.1097/00003081-199803000-00011 9504224

[B4] Ray WA O'Day DM Statistical analysis of multi-eye data in ophthalmic research Investigative Ophthalmology & Visual Science 1985 26 8 1186 1188 4019113

[B5] Barbeito R Herse PR Problem of between-eye correlation for statistical hypothesis testing: rabbit corneal thickness Optometry & Vision Science 1991 68 1 73 76 10.1097/00006324-199101000-00011 2023719

[B6] Katz J Zeger S Liang KY Appropriate statistical methods to account for similarities in binary outcomes between fellow eyes[see comment] Investigative Ophthalmology & Visual Science 1994 35 5 2461 2465 8163336

[B7] Murdoch IE Morris SS Cousens SN People and eyes: statistical approaches in ophthalmology British Journal of Ophthalmology 1998 82 8 971 973 9828786 10.1136/bjo.82.8.971 PMC1722711

[B8] Ananth CV Demissie K Hanley ML Birth weight discordancy and adverse perinatal outcomes among twin gestations in the United States: the effect of placental abruption American Journal of Obstetrics & Gynecology 2003 188 4 954 960 10.1067/mob.2003.210 12712093

[B9] Marttila R Kaprio J Hallman M Respiratory distress syndrome in twin infants compared with singletons American Journal of Obstetrics & Gynecology 2004 191 1 271 276 10.1016/j.ajog.2003.11.020 15295378

[B10] Vergani P Locatelli A Ratti M Scian A Pozzi E Pezzullo JC Ghidini A Preterm twins: what threshold of birth weight discordance heralds major adverse neonatal outcome? American Journal of Obstetrics & Gynecology 2004 191 4 1441 1445 10.1016/j.ajog.2004.05.053 15507980

[B11] Visscher PM Power of the classical twin design revisited Twin Research 2004 7 5 505 512 10.1375/1369052042335250 15527666

[B12] Fanaroff AA Korones SB Wright LL Wright EC Poland RL Bauer CB Tyson JE Philips JB 3rd Edwards W Lucey JF A controlled trial of intravenous immune globulin to reduce nosocomial infections in very-low-birth-weight infants. National Institute of Child Health and Human Development Neonatal Research Network New England Journal of Medicine 1994 330 16 1107 1113 10.1056/NEJM199404213301602 8133853

[B13] Gates S Brocklehurst P How should randomised trials including multiple pregnancies be analysed? BJOG: An International Journal of Obstetrics & Gynaecology 2004 111 3 213 219 10.1111/j.1471-0528.2004.00059.x 14961881

[B14] Laird NM Ware JH Random-effects models for longitudinal data Biometrics 1982 38 4 963 974 10.2307/2529876 7168798

[B15] McCullagh P Nelder JA Generalized Linear Models 1989 2 London: Chapman and Hall

[B16] Breslow NE Clayton DG Approximate inference in generalized linear mixed models Journal of the American Statistical Association 1993 88 9 25 10.2307/2290687

[B17] Molenberghs G Verbeke G Models for Discrete Longitudinal Data 2005 New York: Springer

[B18] Hedeker D Gibbons RD Longitudinal Data Analysis 2006 Hoboken: Wiley

[B19] Liang KY Zeger SL Longitudinal data analysis using generalized linear models Biometrika 1986 73 13 22 10.1093/biomet/73.1.13

[B20] Zeger SL Liang KY Longitudinal data analysis for discrete and continuous outcomes Biometrics 1986 42 1 121 130 10.2307/2531248 3719049

[B21] Diggle PJ Heagerty P Liang KY Zeger SL Analysis of Longitudinal Data 2002 2 Oxford: Oxford University Press

[B22] Glynn RJ Rosner B Accounting for the correlation between fellow eyes in regression analysis Archives of Ophthalmology 1992 110 3 381 387 1543458 10.1001/archopht.1992.01080150079033

[B23] Glynn RJ Rosner B Comparison of alternative regression models for paired binary data Statistics in Medicine 1994 13 10 1023 1036 10.1002/sim.4780131005 8073198

[B24] Leite ML Nicolosi A Statistical analysis of correlated binary data in ophthalmology: a weighted logistic regression approach Ophthalmic Epidemiology 1998 5 3 117 131 10.1076/opep.5.3.117.8365 9805345

[B25] Lindstrom MJ Bates DM Newton-Raphson and EM algorithms for linear mixed-effects models for repeated-measures data Journal of the American Statistical Association 1988 83 1014 1022 10.2307/2290128

[B26] Pinheiro JC Bates DM Approximations to the log-likelihood function in the nonlinear mixed-effects model Journal of Computational and Graphical Statistics 1995 4 12 35 10.2307/1390625

[B27] Rosner B Multivariate methods in ophthalmology with application to other paired-data situations Biometrics 1984 40 4 1025 1035 10.2307/2531153 6534406

