# Supplementary material for: Analysis of neonatal clinical trials with twin births
Source: BMC Med Res Methodol. 2009 Feb 26;9:12. doi: 10.1186/1471-2288-9-12 (PMC2676314; doi:10.1186/1471-2288-9-12)
Supplement: Additional file 1 — Supplementary Table1. Selected simulation results for treatment effect hypothesis testing for continuous outcomes. [file 1471-2288-9-12-S1.doc]

**Supplementary Table 1 - Selected simulation results for treatment effect hypothesis testing for continuous outcomes**

| Simulation parameter | | | | | | Operating characteristic | | | |
| --- | --- | --- | --- | --- | --- | --- | --- | --- | --- |
|  |  |  |  |  |  |  |  | 95% Confidence interval | |
| Randomization | Sample size | Proportion of twins | Effect size | Within-birth correlation | Method | Median bias | Mean squared error | Coverage | Median width |
| Same | 250 | 0.10 | 0 | 0 | ANOVA | -1.38E-4 | 7.16E-3 | 0.949 | 0.332 |
|  |  |  |  |  | LMEM | -1.23E-4 | 7.29E-3 | 0.951 | 0.334 |
|  |  |  |  |  | GEE | -6.69E-5 | 7.55E-3 | 0.944 | 0.328 |
|  |  |  |  | 0.5 | ANOVA | 6.10E-4 | 7.55E-3 | 0.944 | 0.332 |
|  |  |  |  |  | LMEM | 6.24E-4 | 7.44E-3 | 0.949 | 0.338 |
|  |  |  |  |  | GEE | 5.65E-4 | 7.46E-3 | 0.945 | 0.333 |
|  |  |  | 1.5 | 0 | ANOVA | -7.57E-5 | 7.16E-3 | 0.949 | 0.332 |
|  |  |  |  |  | LMEM | -1.21E-4 | 7.29E-3 | 0.951 | 0.334 |
|  |  |  |  |  | GEE | -6.29E-5 | 7.55E-3 | 0.944 | 0.328 |
|  |  |  |  | 0.5 | ANOVA | 6.13E-4 | 7.55E-3 | 0.944 | 0.332 |
|  |  |  |  |  | LMEM | 6.44E-4 | 7.44E-3 | 0.949 | 0.338 |
|  |  |  |  |  | GEE | 6.10E-4 | 7.46E-3 | 0.945 | 0.333 |
|  |  | 0.20 | 0 | 0 | ANOVA | -2.34E-4 | 7.19E-3 | 0.950 | 0.332 |
|  |  |  |  |  | LMEM | -2.56E-4 | 7.24E-3 | 0.952 | 0.333 |
|  |  |  |  |  | GEE | -1.71E-4 | 7.25E-3 | 0.946 | 0.328 |
|  |  |  |  | 0.5 | ANOVA | -5.34E-4 | 7.92E-3 | 0.940 | 0.332 |
|  |  |  |  |  | LMEM | -1.25E-4 | 7.72E-3 | 0.951 | 0.344 |
|  |  |  |  |  | GEE | 9.25E-5 | 7.72E-3 | 0.948 | 0.339 |
|  |  |  | 1.5 | 0 | ANOVA | -2.34E-4 | 7.19E-3 | 0.950 | 0.332 |
|  |  |  |  |  | LMEM | -5.53E-5 | 7.24E-3 | 0.952 | 0.333 |
|  |  |  |  |  | GEE | -3.61E-5 | 7.25E-3 | 0.946 | 0.328 |
|  |  |  |  | 0.5 | ANOVA | -5.15E-4 | 7.92E-3 | 0.941 | 0.332 |
|  |  |  |  |  | LMEM | -9.01E-5 | 7.72E-3 | 0.952 | 0.344 |
|  |  |  |  |  | GEE | 1.61E-4 | 7.72E-3 | 0.948 | 0.339 |
|  | 500 | 0.10 | 0 | 0 | ANOVA | -5.40E-4 | 3.61E-3 | 0.947 | 0.234 |
|  |  |  |  |  | LMEM | -9.08E-4 | 3.63E-3 | 0.948 | 0.235 |
|  |  |  |  |  | GEE | -9.02E-4 | 3.63E-3 | 0.946 | 0.233 |
|  |  |  |  | 0.5 | ANOVA | -8.64E-4 | 3.78E-3 | 0.944 | 0.234 |
|  |  |  |  |  | LMEM | -4.83E-4 | 3.72E-3 | 0.950 | 0.238 |
|  |  |  |  |  | GEE | -6.75E-4 | 3.73E-3 | 0.947 | 0.236 |
|  |  |  | 1.5 | 0 | ANOVA | -5.24E-4 | 3.61E-3 | 0.947 | 0.234 |
|  |  |  |  |  | LMEM | -9.05E-4 | 3.62E-3 | 0.948 | 0.235 |
|  |  |  |  |  | GEE | -8.76E-4 | 3.63E-3 | 0.946 | 0.233 |
|  |  |  |  | 0.5 | ANOVA | -8.68E-4 | 3.78E-3 | 0.944 | 0.234 |
|  |  |  |  |  | LMEM | -4.96E-4 | 3.72E-3 | 0.950 | 0.238 |
|  |  |  |  |  | GEE | -6.93E-4 | 3.72E-3 | 0.947 | 0.236 |
|  |  | 0.20 | 0 | 0 | ANOVA | 4.99E-4 | 3.63E-3 | 0.946 | 0.234 |
|  |  |  |  |  | LMEM | 2.08E-4 | 3.65E-3 | 0.946 | 0.235 |
|  |  |  |  |  | GEE | 2.72E-4 | 3.65E-3 | 0.945 | 0.233 |
|  |  |  |  | 0.5 | ANOVA | 3.12E-4 | 3.99E-3 | 0.935 | 0.234 |
|  |  |  |  |  | LMEM | 1.47E-4 | 3.88E-3 | 0.947 | 0.242 |
|  |  |  |  |  | GEE | -1.04E-4 | 3.88E-3 | 0.945 | 0.241 |
|  |  |  | 1.5 | 0 | ANOVA | 4.56E-4 | 3.63E-3 | 0.946 | 0.234 |
|  |  |  |  |  | LMEM | 1.75E-4 | 3.65E-3 | 0.947 | 0.235 |
|  |  |  |  |  | GEE | 2.25E-4 | 3.65E-3 | 0.945 | 0.233 |
|  |  |  |  | 0.5 | ANOVA | 3.19E-4 | 3.99E-3 | 0.935 | 0.234 |
|  |  |  |  |  | LMEM | 1.47E-4 | 3.88E-3 | 0.947 | 0.242 |
|  |  |  |  |  | GEE | -1.04E-4 | 3.88E-3 | 0.945 | 0.241 |
| Independent | 250 | 0.10 | 0 | 0 | ANOVA | 1.65E-4 | 7.01E-3 | 0.950 | 0.331 |
|  |  |  |  |  | LMEM | 4.14E-4 | 7.09E-3 | 0.950 | 0.334 |
|  |  |  |  |  | GEE | 3.18E-4 | 7.60E-3 | 0.944 | 0.328 |
|  |  |  |  | 0.5 | ANOVA | -1.71E-4 | 7.14E-3 | 0.951 | 0.331 |
|  |  |  |  |  | LMEM | -1.33E-4 | 7.00E-3 | 0.954 | 0.329 |
|  |  |  |  |  | GEE | 1.17E-3 | 1.39E-2 | 0.937 | 0.328 |
|  |  |  | 1.5 | 0 | ANOVA | 1.08E-4 | 7.01E-3 | 0.950 | 0.331 |
|  |  |  |  |  | LMEM | 3.78E-4 | 7.09E-3 | 0.950 | 0.334 |
|  |  |  |  |  | GEE | 2.98E-4 | 7.60E-3 | 0.944 | 0.328 |
|  |  |  |  | 0.5 | ANOVA | -2.15E-4 | 7.14E-3 | 0.951 | 0.331 |
|  |  |  |  |  | LMEM | -1.81E-4 | 7.00E-3 | 0.954 | 0.329 |
|  |  |  |  |  | GEE | 1.13E-3 | 1.39E-2 | 0.937 | 0.328 |
|  |  | 0.20 | 0 | 0 | ANOVA | 5.81E-4 | 7.15E-3 | 0.949 | 0.331 |
|  |  |  |  |  | LMEM | 9.73E-4 | 7.23E-3 | 0.950 | 0.333 |
|  |  |  |  |  | GEE | 1.08E-3 | 7.24E-3 | 0.945 | 0.327 |
|  |  |  |  | 0.5 | ANOVA | -1.86E-3 | 7.22E-3 | 0.948 | 0.331 |
|  |  |  |  |  | LMEM | -1.36E-3 | 6.82E-3 | 0.950 | 0.324 |
|  |  |  |  |  | GEE | -6.48E-4 | 7.74E-3 | 0.943 | 0.320 |
|  |  |  | 1.5 | 0 | ANOVA | 5.38E-4 | 7.15E-3 | 0.949 | 0.331 |
|  |  |  |  |  | LMEM | 9.47E-4 | 7.23E-3 | 0.950 | 0.333 |
|  |  |  |  |  | GEE | 1.03E-3 | 7.24E-3 | 0.945 | 0.327 |
|  |  |  |  | 0.5 | ANOVA | -1.84E-3 | 7.22E-3 | 0.948 | 0.331 |
|  |  |  |  |  | LMEM | -1.32E-3 | 6.82E-3 | 0.950 | 0.324 |
|  |  |  |  |  | GEE | -6.21E-4 | 7.74E-3 | 0.943 | 0.320 |
|  | 500 | 0.10 | 0 | 0 | ANOVA | -1.38E-4 | 3.49E-3 | 0.951 | 0.234 |
|  |  |  |  |  | LMEM | -1.52E-4 | 3.51E-3 | 0.951 | 0.235 |
|  |  |  |  |  | GEE | -3.69E-4 | 3.51E-3 | 0.950 | 0.232 |
|  |  |  |  | 0.5 | ANOVA | 5.18E-4 | 3.51E-3 | 0.953 | 0.234 |
|  |  |  |  |  | LMEM | -8.96E-5 | 3.44E-3 | 0.953 | 0.231 |
|  |  |  |  |  | GEE | 2.24E-4 | 4.78E-3 | 0.945 | 0.231 |
|  |  |  | 1.5 | 0 | ANOVA | -1.19E-4 | 3.49E-3 | 0.951 | 0.234 |
|  |  |  |  |  | LMEM | -1.45E-4 | 3.51E-3 | 0.951 | 0.235 |
|  |  |  |  |  | GEE | -3.47E-4 | 3.51E-3 | 0.950 | 0.232 |
|  |  |  |  | 0.5 | ANOVA | 5.22E-4 | 3.52E-3 | 0.953 | 0.234 |
|  |  |  |  |  | LMEM | -8.96E-5 | 3.43E-3 | 0.953 | 0.231 |
|  |  |  |  |  | GEE | 2.33E-4 | 4.77E-3 | 0.945 | 0.231 |
|  |  | 0.20 | 0 | 0 | ANOVA | -5.24E-4 | 3.45E-3 | 0.951 | 0.234 |
|  |  |  |  |  | LMEM | -7.01E-4 | 3.46E-3 | 0.951 | 0.234 |
|  |  |  |  |  | GEE | -7.22E-4 | 3.46E-3 | 0.950 | 0.232 |
|  |  |  |  | 0.5 | ANOVA | 5.04E-4 | 3.53E-3 | 0.948 | 0.234 |
|  |  |  |  |  | LMEM | 8.65E-4 | 3.35E-3 | 0.947 | 0.227 |
|  |  |  |  |  | GEE | 8.24E-4 | 3.46E-3 | 0.944 | 0.226 |
|  |  |  | 1.5 | 0 | ANOVA | -5.51E-4 | 3.45E-3 | 0.951 | 0.234 |
|  |  |  |  |  | LMEM | -7.21E-4 | 3.46E-3 | 0.951 | 0.234 |
|  |  |  |  |  | GEE | -7.39E-4 | 3.46E-3 | 0.950 | 0.232 |
|  |  |  |  | 0.5 | ANOVA | 5.04E-4 | 3.53E-3 | 0.948 | 0.234 |
|  |  |  |  |  | LMEM | 8.64E-4 | 3.35E-3 | 0.947 | 0.227 |
|  |  |  |  |  | GEE | 7.96E-4 | 3.46E-3 | 0.944 | 0.226 |
| Opposite | 250 | 0.10 | 0 | 0 | ANOVA | -6.62E-4 | 7.24E-3 | 0.949 | 0.332 |
|  |  |  |  |  | LMEM | -2.00E-4 | 7.31E-3 | 0.947 | 0.334 |
|  |  |  |  |  | GEE | -5.10E-4 | 7.54E-3 | 0.943 | 0.329 |
|  |  |  |  | 0.5 | ANOVA | 1.32E-3 | 6.71E-3 | 0.958 | 0.332 |
|  |  |  |  |  | LMEM | 1.68E-3 | 6.57E-3 | 0.952 | 0.321 |
|  |  |  |  |  | GEE | 1.90E-3 | 1.04E-2 | 0.942 | 0.323 |
|  |  |  | 1.5 | 0 | ANOVA | -6.62E-4 | 7.24E-3 | 0.949 | 0.332 |
|  |  |  |  |  | LMEM | -1.66E-4 | 7.31E-3 | 0.947 | 0.334 |
|  |  |  |  |  | GEE | -5.04E-4 | 7.54E-3 | 0.943 | 0.329 |
|  |  |  |  | 0.5 | ANOVA | 1.39E-3 | 6.71E-3 | 0.958 | 0.332 |
|  |  |  |  |  | LMEM | 1.74E-3 | 6.58E-3 | 0.952 | 0.321 |
|  |  |  |  |  | GEE | 1.94E-3 | 1.04E-2 | 0.942 | 0.323 |
|  |  | 0.20 | 0 | 0 | ANOVA | 5.69E-4 | 7.19E-3 | 0.948 | 0.332 |
|  |  |  |  |  | LMEM | 2.68E-4 | 7.26E-3 | 0.948 | 0.334 |
|  |  |  |  |  | GEE | 3.15E-5 | 7.27E-3 | 0.944 | 0.328 |
|  |  |  |  | 0.5 | ANOVA | 8.05E-4 | 6.35E-3 | 0.964 | 0.332 |
|  |  |  |  |  | LMEM | 4.97E-4 | 6.02E-3 | 0.953 | 0.307 |
|  |  |  |  |  | GEE | 2.62E-4 | 6.68E-3 | 0.946 | 0.306 |
|  |  |  | 1.5 | 0 | ANOVA | 5.88E-4 | 7.19E-3 | 0.948 | 0.332 |
|  |  |  |  |  | LMEM | 2.76E-4 | 7.25E-3 | 0.948 | 0.334 |
|  |  |  |  |  | GEE | 5.19E-5 | 7.27E-3 | 0.944 | 0.328 |
|  |  |  |  | 0.5 | ANOVA | 8.38E-4 | 6.35E-3 | 0.964 | 0.332 |
|  |  |  |  |  | LMEM | 5.09E-4 | 6.02E-3 | 0.953 | 0.307 |
|  |  |  |  |  | GEE | 3.42E-4 | 6.68E-3 | 0.946 | 0.306 |
|  | 500 | 0.10 | 0 | 0 | ANOVA | 1.38E-3 | 3.59E-3 | 0.951 | 0.234 |
|  |  |  |  |  | LMEM | 1.23E-3 | 3.61E-3 | 0.951 | 0.235 |
|  |  |  |  |  | GEE | 1.08E-3 | 3.61E-3 | 0.949 | 0.233 |
|  |  |  |  | 0.5 | ANOVA | 9.61E-4 | 3.40E-3 | 0.957 | 0.234 |
|  |  |  |  |  | LMEM | 7.33E-4 | 3.32E-3 | 0.949 | 0.225 |
|  |  |  |  |  | GEE | 9.44E-4 | 4.02E-3 | 0.946 | 0.226 |
|  |  |  | 1.5 | 0 | ANOVA | 1.39E-3 | 3.59E-3 | 0.951 | 0.234 |
|  |  |  |  |  | LMEM | 1.27E-3 | 3.60E-3 | 0.951 | 0.235 |
|  |  |  |  |  | GEE | 1.12E-3 | 3.61E-3 | 0.949 | 0.233 |
|  |  |  |  | 0.5 | ANOVA | 9.80E-4 | 3.40E-3 | 0.957 | 0.234 |
|  |  |  |  |  | LMEM | 7.33E-4 | 3.31E-3 | 0.949 | 0.225 |
|  |  |  |  |  | GEE | 9.44E-4 | 4.02E-3 | 0.946 | 0.226 |
|  |  | 0.20 | 0 | 0 | ANOVA | 6.66E-4 | 3.58E-3 | 0.951 | 0.234 |
|  |  |  |  |  | LMEM | 6.60E-4 | 3.58E-3 | 0.952 | 0.235 |
|  |  |  |  |  | GEE | 6.94E-4 | 3.58E-3 | 0.949 | 0.233 |
|  |  |  |  | 0.5 | ANOVA | 6.09E-6 | 3.24E-3 | 0.962 | 0.234 |
|  |  |  |  |  | LMEM | 6.27E-4 | 3.00E-3 | 0.951 | 0.215 |
|  |  |  |  |  | GEE | 2.37E-4 | 3.07E-3 | 0.951 | 0.215 |
|  |  |  | 1.5 | 0 | ANOVA | 6.66E-4 | 3.58E-3 | 0.951 | 0.234 |
|  |  |  |  |  | LMEM | 6.60E-4 | 3.58E-3 | 0.952 | 0.235 |
|  |  |  |  |  | GEE | 6.94E-4 | 3.58E-3 | 0.949 | 0.233 |
|  |  |  |  | 0.5 | ANOVA | 2.11E-5 | 3.24E-3 | 0.962 | 0.234 |
|  |  |  |  |  | LMEM | 6.67E-4 | 3.00E-3 | 0.951 | 0.215 |
|  |  |  |  |  | GEE | 2.59E-4 | 3.07E-3 | 0.951 | 0.215 |
