# Supplementary material for: Analysis of neonatal clinical trials with twin births
Source: BMC Med Res Methodol. 2009 Feb 26;9:12. doi: 10.1186/1471-2288-9-12 (PMC2676314; doi:10.1186/1471-2288-9-12)
Supplement: Additional file 2 — Supplementary Table2. Selected simulation results for treatment effect hypothesis testing for binary outcomes. [file 1471-2288-9-12-S2.doc]

**Supplementary Table 2 - Selected simulation results for treatment effect hypothesis testing for binary outcomes**

| Simulation parameter | | | | | | Operating characteristic | | | |
| --- | --- | --- | --- | --- | --- | --- | --- | --- | --- |
|  |  |  |  |  |  |  |  | 95% Confidence interval | |
| Randomization | Sample size | Proportion of twins | Effect size | Within-birth correlation | Method | Median bias | Mean squared error | Coverage | Median width |
| Same | 250 | 0.10 | -1 | -2 | LOGISTIC | 0.008 | 0.016 | 0.954 | 0.492 |
|  |  |  |  |  | GLMM | 0.007 | 0.016 | 0.946 | 0.466 |
|  |  |  |  |  | GEE | 0.017 | 0.018 | 0.943 | 0.497 |
|  |  |  |  | -1 | LOGISTIC | 0.003 | 0.016 | 0.953 | 0.493 |
|  |  |  |  |  | GLMM | 0.003 | 0.016 | 0.944 | 0.467 |
|  |  |  |  |  | GEE | 0.007 | 0.017 | 0.947 | 0.495 |
|  |  |  | 0 | -2 | LOGISTIC | <0.001 | 0.080 | 0.957 | 1.109 |
|  |  |  |  |  | GLMM | <0.001 | 0.080 | 0.947 | 1.068 |
|  |  |  |  |  | GEE | -0.003 | 0.081 | 0.954 | 1.077 |
|  |  |  |  | -1 | LOGISTIC | <0.001 | 0.083 | 0.955 | 1.122 |
|  |  |  |  |  | GLMM | <0.001 | 0.084 | 0.943 | 1.083 |
|  |  |  |  |  | GEE | -0.002 | 0.085 | 0.951 | 1.102 |
|  |  | 0.20 | -1 | -2 | LOGISTIC | 0.019 | 0.017 | 0.953 | 0.504 |
|  |  |  |  |  | GLMM | 0.019 | 0.017 | 0.956 | 0.479 |
|  |  |  |  |  | GEE | 0.030 | 0.018 | 0.940 | 0.498 |
|  |  |  |  | -1 | LOGISTIC | 0.010 | 0.017 | 0.952 | 0.501 |
|  |  |  |  |  | GLMM | 0.009 | 0.017 | 0.944 | 0.475 |
|  |  |  |  |  | GEE | 0.012 | 0.017 | 0.946 | 0.495 |
|  |  |  | 0 | -2 | LOGISTIC | <0.001 | 0.080 | 0.956 | 1.111 |
|  |  |  |  |  | GLMM | <0.001 | 0.080 | 0.950 | 1.071 |
|  |  |  |  |  | GEE | 0.005 | 0.078 | 0.946 | 1.049 |
|  |  |  |  | -1 | LOGISTIC | 0.001 | 0.084 | 0.956 | 1.124 |
|  |  |  |  |  | GLMM | 0.001 | 0.084 | 0.947 | 1.084 |
|  |  |  |  |  | GEE | 0.005 | 0.083 | 0.948 | 1.091 |
|  | 500 | 0.10 | -1 | -2 | LOGISTIC | 0.007 | 0.007 | 0.953 | 0.336 |
|  |  |  |  |  | GLMM | 0.007 | 0.007 | 0.955 | 0.328 |
|  |  |  |  |  | GEE | 0.013 | 0.008 | 0.945 | 0.335 |
|  |  |  |  | -1 | LOGISTIC | 0.002 | 0.007 | 0.950 | 0.338 |
|  |  |  |  |  | GLMM | 0.002 | 0.007 | 0.952 | 0.328 |
|  |  |  |  |  | GEE | 0.004 | 0.008 | 0.947 | 0.336 |
|  |  |  | 0 | -2 | LOGISTIC | -0.005 | 0.038 | 0.953 | 0.764 |
|  |  |  |  |  | GLMM | -0.005 | 0.038 | 0.952 | 0.750 |
|  |  |  |  |  | GEE | -0.004 | 0.038 | 0.948 | 0.741 |
|  |  |  |  | -1 | LOGISTIC | -0.006 | 0.040 | 0.952 | 0.769 |
|  |  |  |  |  | GLMM | -0.006 | 0.040 | 0.948 | 0.757 |
|  |  |  |  |  | GEE | -0.006 | 0.040 | 0.947 | 0.758 |
|  |  | 0.20 | -1 | -2 | LOGISTIC | 0.018 | 0.008 | 0.948 | 0.343 |
|  |  |  |  |  | GLMM | 0.018 | 0.008 | 0.962 | 0.334 |
|  |  |  |  |  | GEE | 0.026 | 0.009 | 0.933 | 0.338 |
|  |  |  |  | -1 | LOGISTIC | 0.009 | 0.008 | 0.952 | 0.341 |
|  |  |  |  |  | GLMM | 0.009 | 0.008 | 0.955 | 0.332 |
|  |  |  |  |  | GEE | 0.011 | 0.008 | 0.946 | 0.337 |
|  |  |  | 0 | -2 | LOGISTIC | <0.001 | 0.036 | 0.960 | 0.766 |
|  |  |  |  |  | GLMM | <0.001 | 0.036 | 0.958 | 0.751 |
|  |  |  |  |  | GEE | -0.001 | 0.035 | 0.950 | 0.725 |
|  |  |  |  | -1 | LOGISTIC | -0.001 | 0.037 | 0.959 | 0.772 |
|  |  |  |  |  | GLMM | -0.001 | 0.037 | 0.955 | 0.757 |
|  |  |  |  |  | GEE | -0.001 | 0.037 | 0.952 | 0.751 |
| Independent | 250 | 0.10 | -1 | -2 | LOGISTIC | -0.005 | 0.015 | 0.950 | 0.475 |
|  |  |  |  |  | GLMM | -0.006 | 0.015 | 0.929 | 0.449 |
|  |  |  |  |  | GEE | 0.001 | 0.027 | 0.943 | 0.483 |
|  |  |  |  | -1 | LOGISTIC | -0.005 | 0.016 | 0.953 | 0.484 |
|  |  |  |  |  | GLMM | -0.005 | 0.016 | 0.931 | 0.455 |
|  |  |  |  |  | GEE | -0.002 | 0.017 | 0.949 | 0.485 |
|  |  |  | 0 | -2 | LOGISTIC | <0.001 | 0.086 | 0.947 | 1.108 |
|  |  |  |  |  | GLMM | <0.001 | 0.086 | 0.937 | 1.067 |
|  |  |  |  |  | GEE | -0.002 | 0.097 | 0.946 | 1.097 |
|  |  |  |  | -1 | LOGISTIC | <0.001 | 0.088 | 0.947 | 1.121 |
|  |  |  |  |  | GLMM | <0.001 | 0.089 | 0.935 | 1.079 |
|  |  |  |  |  | GEE | -0.001 | 0.091 | 0.945 | 1.110 |
|  |  | 0.20 | -1 | -2 | LOGISTIC | -0.005 | 0.015 | 0.950 | 0.474 |
|  |  |  |  |  | GLMM | -0.005 | 0.015 | 0.930 | 0.449 |
|  |  |  |  |  | GEE | 0.003 | 0.016 | 0.945 | 0.476 |
|  |  |  |  | -1 | LOGISTIC | -0.005 | 0.016 | 0.950 | 0.482 |
|  |  |  |  |  | GLMM | -0.006 | 0.016 | 0.932 | 0.455 |
|  |  |  |  |  | GEE | -0.002 | 0.016 | 0.947 | 0.482 |
|  |  |  | 0 | -2 | LOGISTIC | <0.001 | 0.085 | 0.949 | 1.103 |
|  |  |  |  |  | GLMM | <0.001 | 0.085 | 0.937 | 1.062 |
|  |  |  |  |  | GEE | -0.006 | 0.084 | 0.946 | 1.080 |
|  |  |  |  | -1 | LOGISTIC | <0.001 | 0.088 | 0.949 | 1.114 |
|  |  |  |  |  | GLMM | <0.001 | 0.089 | 0.937 | 1.073 |
|  |  |  |  |  | GEE | -0.007 | 0.088 | 0.945 | 1.099 |
|  | 500 | 0.10 | -1 | -2 | LOGISTIC | -0.002 | 0.007 | 0.954 | 0.328 |
|  |  |  |  |  | GLMM | -0.002 | 0.007 | 0.936 | 0.319 |
|  |  |  |  |  | GEE | 0.002 | 0.007 | 0.950 | 0.330 |
|  |  |  |  | -1 | LOGISTIC | -0.003 | 0.007 | 0.953 | 0.333 |
|  |  |  |  |  | GLMM | -0.003 | 0.007 | 0.937 | 0.323 |
|  |  |  |  |  | GEE | -0.002 | 0.007 | 0.951 | 0.333 |
|  |  |  | 0 | -2 | LOGISTIC | <0.001 | 0.039 | 0.952 | 0.762 |
|  |  |  |  |  | GLMM | <0.001 | 0.039 | 0.943 | 0.749 |
|  |  |  |  |  | GEE | -0.004 | 0.038 | 0.952 | 0.755 |
|  |  |  |  | -1 | LOGISTIC | <0.001 | 0.039 | 0.951 | 0.770 |
|  |  |  |  |  | GLMM | <0.001 | 0.040 | 0.944 | 0.757 |
|  |  |  |  |  | GEE | -0.002 | 0.040 | 0.950 | 0.767 |
|  |  | 0.20 | -1 | -2 | LOGISTIC | -0.002 | 0.007 | 0.958 | 0.327 |
|  |  |  |  |  | GLMM | -0.002 | 0.007 | 0.941 | 0.318 |
|  |  |  |  |  | GEE | 0.006 | 0.007 | 0.952 | 0.329 |
|  |  |  |  | -1 | LOGISTIC | -0.004 | 0.007 | 0.954 | 0.331 |
|  |  |  |  |  | GLMM | -0.004 | 0.007 | 0.940 | 0.323 |
|  |  |  |  |  | GEE | -0.001 | 0.007 | 0.952 | 0.332 |
|  |  |  | 0 | -2 | LOGISTIC | <0.001 | 0.038 | 0.952 | 0.765 |
|  |  |  |  |  | GLMM | <0.001 | 0.038 | 0.949 | 0.750 |
|  |  |  |  |  | GEE | -0.002 | 0.037 | 0.952 | 0.751 |
|  |  |  |  | -1 | LOGISTIC | <0.001 | 0.039 | 0.953 | 0.772 |
|  |  |  |  |  | GLMM | <0.001 | 0.039 | 0.947 | 0.759 |
|  |  |  |  |  | GEE | -0.002 | 0.039 | 0.952 | 0.766 |
| Opposite | 250 | 0.10 | -1 | -2 | LOGISTIC | -0.010 | 0.014 | 0.950 | 0.468 |
|  |  |  |  |  | GLMM | -0.010 | 0.014 | 0.924 | 0.442 |
|  |  |  |  |  | GEE | -0.006 | 0.015 | 0.948 | 0.469 |
|  |  |  |  | -1 | LOGISTIC | -0.007 | 0.015 | 0.953 | 0.480 |
|  |  |  |  |  | GLMM | -0.008 | 0.015 | 0.924 | 0.452 |
|  |  |  |  |  | GEE | -0.006 | 0.015 | 0.951 | 0.481 |
|  |  |  | 0 | -2 | LOGISTIC | <0.001 | 0.086 | 0.952 | 1.111 |
|  |  |  |  |  | GLMM | <0.001 | 0.087 | 0.940 | 1.070 |
|  |  |  |  |  | GEE | <0.001 | 0.086 | 0.952 | 1.118 |
|  |  |  |  | -1 | LOGISTIC | <0.001 | 0.087 | 0.950 | 1.122 |
|  |  |  |  |  | GLMM | <0.001 | 0.088 | 0.943 | 1.079 |
|  |  |  |  |  | GEE | <0.001 | 0.087 | 0.950 | 1.125 |
|  |  | 0.20 | -1 | -2 | LOGISTIC | -0.021 | 0.014 | 0.945 | 0.457 |
|  |  |  |  |  | GLMM | -0.021 | 0.014 | 0.908 | 0.433 |
|  |  |  |  |  | GEE | -0.016 | 0.014 | 0.948 | 0.464 |
|  |  |  |  | -1 | LOGISTIC | -0.012 | 0.015 | 0.950 | 0.473 |
|  |  |  |  |  | GLMM | -0.013 | 0.015 | 0.920 | 0.446 |
|  |  |  |  |  | GEE | -0.010 | 0.015 | 0.952 | 0.479 |
|  |  |  | 0 | -2 | LOGISTIC | <0.001 | 0.094 | 0.940 | 1.110 |
|  |  |  |  |  | GLMM | <0.001 | 0.094 | 0.938 | 1.070 |
|  |  |  |  |  | GEE | <0.001 | 0.093 | 0.948 | 1.137 |
|  |  |  |  | -1 | LOGISTIC | <0.001 | 0.092 | 0.946 | 1.117 |
|  |  |  |  |  | GLMM | <0.001 | 0.093 | 0.938 | 1.075 |
|  |  |  |  |  | GEE | -0.002 | 0.093 | 0.949 | 1.132 |
|  | 500 | 0.10 | -1 | -2 | LOGISTIC | -0.012 | 0.007 | 0.947 | 0.320 |
|  |  |  |  |  | GLMM | -0.012 | 0.007 | 0.928 | 0.312 |
|  |  |  |  |  | GEE | -0.009 | 0.007 | 0.948 | 0.323 |
|  |  |  |  | -1 | LOGISTIC | -0.008 | 0.007 | 0.948 | 0.328 |
|  |  |  |  |  | GLMM | -0.008 | 0.007 | 0.930 | 0.318 |
|  |  |  |  |  | GEE | -0.007 | 0.007 | 0.948 | 0.330 |
|  |  |  | 0 | -2 | LOGISTIC | <0.001 | 0.040 | 0.950 | 0.766 |
|  |  |  |  |  | GLMM | <0.001 | 0.040 | 0.941 | 0.752 |
|  |  |  |  |  | GEE | -0.002 | 0.040 | 0.951 | 0.774 |
|  |  |  |  | -1 | LOGISTIC | <0.001 | 0.041 | 0.949 | 0.772 |
|  |  |  |  |  | GLMM | <0.001 | 0.041 | 0.940 | 0.758 |
|  |  |  |  |  | GEE | <0.001 | 0.041 | 0.950 | 0.777 |
|  |  | 0.20 | -1 | -2 | LOGISTIC | -0.021 | 0.007 | 0.941 | 0.311 |
|  |  |  |  |  | GLMM | -0.021 | 0.007 | 0.908 | 0.303 |
|  |  |  |  |  | GEE | -0.016 | 0.007 | 0.946 | 0.317 |
|  |  |  |  | -1 | LOGISTIC | -0.013 | 0.007 | 0.946 | 0.323 |
|  |  |  |  |  | GLMM | -0.013 | 0.007 | 0.931 | 0.314 |
|  |  |  |  |  | GEE | -0.012 | 0.007 | 0.949 | 0.327 |
|  |  |  | 0 | -2 | LOGISTIC | -0.002 | 0.040 | 0.948 | 0.764 |
|  |  |  |  |  | GLMM | -0.002 | 0.040 | 0.941 | 0.749 |
|  |  |  |  |  | GEE | -0.003 | 0.040 | 0.954 | 0.783 |
|  |  |  |  | -1 | LOGISTIC | <0.001 | 0.040 | 0.952 | 0.771 |
|  |  |  |  |  | GLMM | <0.001 | 0.040 | 0.942 | 0.756 |
|  |  |  |  |  | GEE | -0.002 | 0.040 | 0.955 | 0.783 |
